# Accurate Imputation of Greenhouse Environment Data for Data Integrity Utilizing Two-Dimensional Convolutional Neural Networks

**DOI:** 10.3390/s21062187

**Published:** 2021-03-20

**Authors:** Taewon Moon, Joon Woo Lee, Jung Eek Son

**Affiliations:** 1 Department of Agriculture, Forestry and Bioresources, Seoul National University, Seoul 08826, Korea; ataraxno@snu.ac.kr; 2 Department of Smart Agriculture, Jeonju University, Jeonju 55069, Korea; jweee2@jj.ac.kr; 3 Research Institute of Agriculture and Life Sciences, Seoul National University, Seoul 08826, Korea

**Keywords:** artificial intelligence, deep learning, interpolation, machine learning, plant environment

## Abstract

Greenhouses require accurate and reliable data to interpret the microclimate and maximize resource use efficiency. However, greenhouse conditions are harsh for electrical sensors collecting environmental data. Convolutional neural networks (ConvNets) enable complex interpretation by multiplying the input data. The objective of this study was to impute missing tabular data collected from several greenhouses using a ConvNet architecture called U-Net. Various data-loss conditions with errors in individual sensors and in all sensors were assumed. The U-Net with a screen size of 50 exhibited the highest coefficient of determination values and the lowest root-mean-square errors for all environmental factors used in this study. U-Net_50_ correctly learned the changing patterns of the greenhouse environment from the training dataset. Therefore, the U-Net architecture can be used for the imputation of tabular data in greenhouses if the model is correctly trained. Growers can secure data integrity with imputed data, which could increase crop productivity and quality in greenhouses.

## 1. Introduction

Agricultural systems and their models vary across spatial and temporal scales [1]. Greenhouses, which represent a small agricultural system, increase the yield and quality of agricultural crops [2]. The greenhouse microclimate is manipulated to reduce energy input and increase crop yield and quality [3,4,5]. Growers’ strategies make distinctive microclimates to maximize resource use efficiency. Therefore, the microclimate is partly or totally anthropogenic in any form of greenhouse.

Since the greenhouse environment should be monitored for precise control, multidimensional information is accumulated and interpreted in different ways [6,7,8]. The accumulated data can explain the interactions between the environment and the crops [9,10]. Recent developments in sensors and algorithms have also allowed machine learning and deep learning to be applied to agricultural data [11].

However, the internal environment of a greenhouse can be harsh for electrical sensors. The greenhouse may be close to water, and high solar radiation could heat the sensors [12]. Root-zone sensors could also be blocked by irrigational problems [13]. In this case, sensors cannot obtain complete data without errors, resulting in low data integrity. In addition, sensors in greenhouses are likely to lose their connection because of various external causes, such as blackouts or floods. Under such conditions, relatively long-term datasets could be lost, which can distort the accumulated environmental data [14]. Because past environments cannot be inferred from distorted data, a method to restore lost data is required.

Because environmental factors in greenhouses influence each other interactively and temporally, complex interpretation should be considered in interpolating environmental data. Convolutional neural networks (ConvNets) enable complex interpretation by multiplying the input data [15]. ConvNets are mainly used for image processing, but they also exhibit high performance in the extraction of interactive features within inputs [16,17]. Therefore, data imputation using a two-dimensional ConvNet can be performed for the obtained greenhouse environmental data. The objective of this study is to impute missing tabular data collected from several greenhouses using a ConvNet.

## 2. Materials and Methods

### 2.1. U-Net Model Architecture and Prediction Workflow

**Figure 1:**
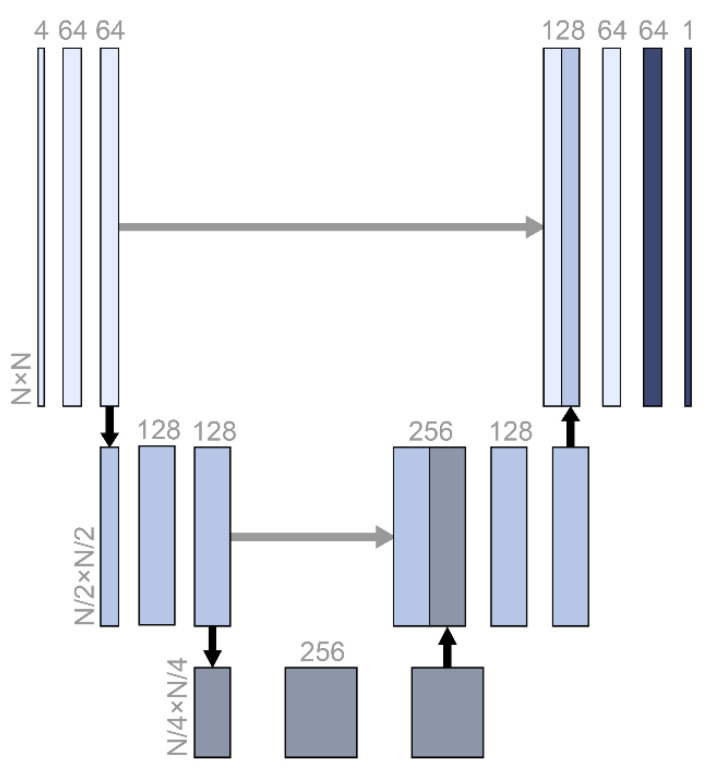
**The** U-Net structure used in this study. N was 5, 10, 20, or 100, which was the same as the screen size and input size. The numbers with horizontal writing represent the dimensions of the relevant vectors. Black and gray arrows represent max pooling and skip connection.

A fully convolutional network architecture called U-Net was used for data imputation (Figure 1). From the original input size, N, the size was compressed to one-quarter, and the abstracted features were restored in stages. U-Net is often used for image segmentation tasks in medical image datasets where the output has similar features and the same size as the input [18]. The architecture was the same as that of vanilla U-Net, which has a skip connection (Figure 1).

Every layer in a neural network algorithm is expected to abstract the relation between the input and output hierarchically [19]. However, the layers could become short-sighted and learn only the relation between the previous and the next layers. This can reduce the model performance, especially when the model should restore the original input size. The skip connection architecture delivers the previous abstraction to the deeper layers directly [20]. In this study of data imputation, not only did the output have to be the same size as the input, but also, the output was largely related with the original input. Therefore, the U-Net architecture was expected to be effective for the data imputation. Zero padding was added to sustain even-numbered inputs for the convolutional layer. The cost function was the mean square error.

### 2.2. Experimental Greenhouse Environmental Data

**Figure 2:**
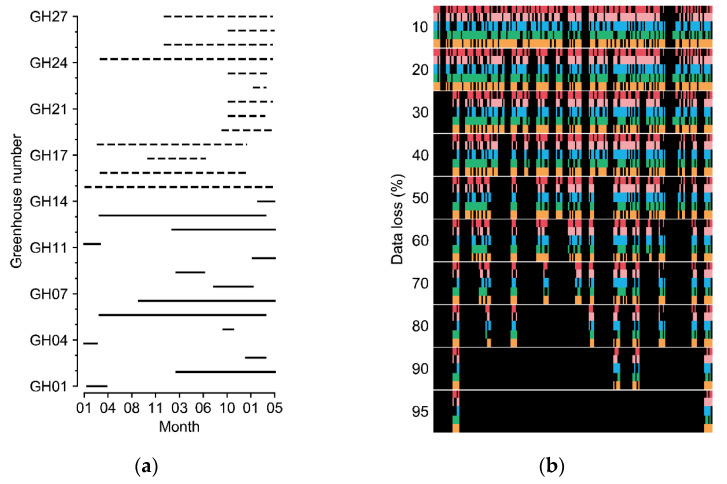
(**a**) Cultivation periods of the greenhouses. Solid and dashed lines represent tomato and sweet pepper greenhouses, respectively. (**b**) Examples of manipulated data loss. Each color from the top represents five target factors of internal temperature, external temperature, internal relative humidity, internal CO_2_ concentration, and radiation. Black blanks represent the data loss. Refer to Table 1 for the units of environmental factors.

Greenhouses cultivating sweet peppers (*Capsicum annuum* L.) and tomatoes (*Solanum lycopersicum* L.) in various regions of South Korea were used to obtain the experimental datasets. The covering materials varied from arch-type plastic to Venlo-type glasses. The minimum and maximum sizes of the sweet pepper greenhouses (width × length × height) were 7 m × 80 m × 5 m and 100 m × 110 m × 5.7 m, respectively; those of the tomato greenhouses were 7 m × 53 m × 3 m and 66 m × 100 m × 4.5 m, respectively. The data collection periods varied according to the greenhouse (Figure 2a).

The data interval was one hour, and the collected environmental factors were internal temperature (T_in_), external temperature (T_out_), internal relative humidity (RH), CO_2_ concentration (CO_2_), and radiation (Rad). The collected data included erroneous values (Table 1).

### 2.3. Manipulation of the Data-Loss Conditions and Data Preprocessing

In this study, incomplete data with errors and short-term losses were used. Outliers of the measured data were deleted, and short-term missing data were linearly interpolated. After processing, the data were considered intact. The data loss was manipulated with the collected environment factors for the experiments (Figure 2b). The random seed for generating random numbers was fixed for the comparisons. Various data-loss conditions with errors in individual sensors and in all sensors were assumed. Losses in all sensors can result from electrical malfunctions such as a blackout, which makes it impossible to refer to other sensor values at the current loss time. The error rates of the individual sensors and all sensors were 30%. Because all-sensor losses usually accompany long-term loss, all-sensor loss times were set to two days (48 data indices). All losses were randomized using a random number generator.

**Figure 3:**
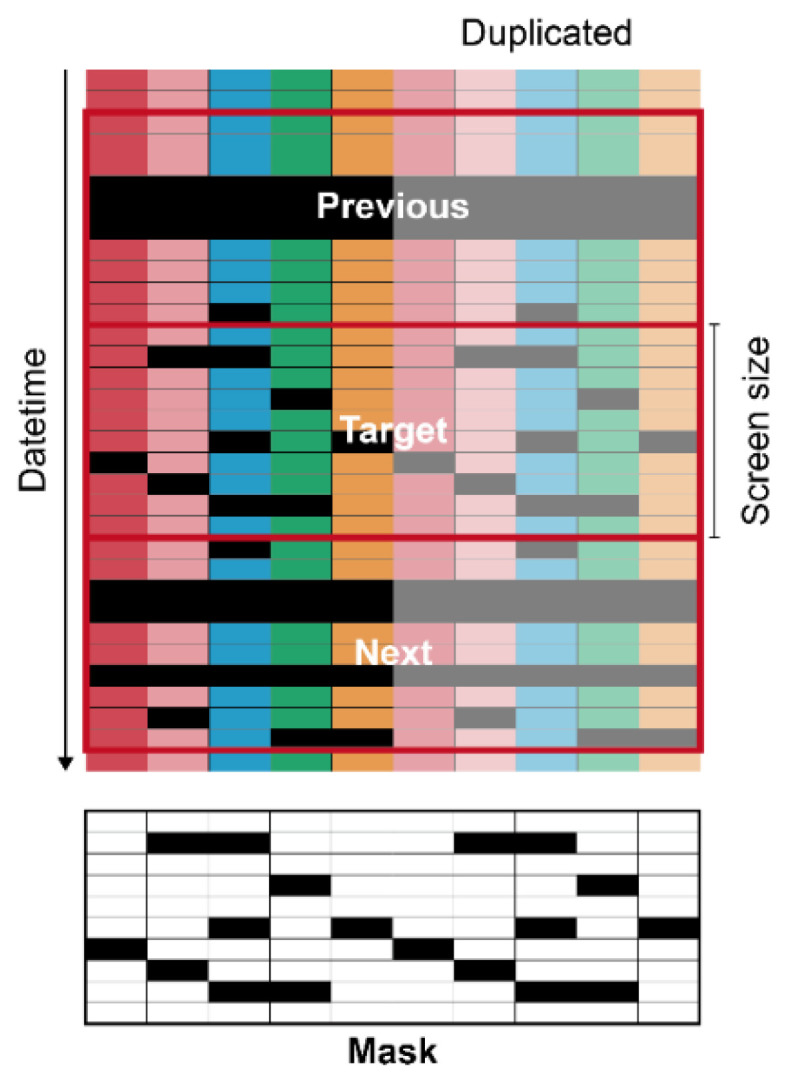
Diagram of data preprocessing for U-Net. Each color represents each environmental factor. Each color from the left to right represents the five target factors of internal temperature, external temperature, internal relative humidity, internal CO_2_ concentration, and radiation. Black cells are missing data. The values in a mask were 0 and 1 for black and white, respectively.

The input matrices had specific screen sizes of 5, 10, 20, and 100 to ensure that they were rectangular (Figure 3).

The screen sizes are represented as subscripts of the model name. Five input features were used; therefore, the input features were duplicated to increase the input size to match the screen size when needed. Consequently, the output also followed the screen sizes, and the duplicated outputs were averaged, except for two outliers in both extremes, expecting a similar effect to the model ensemble. To make the U-Net consider adjacent data, the tabular data in the previous and next date time from the target were used as the input. A mask matrix representing missing values was also added to the input. Intact and missing data were 1 and 0 in the matrix, respectively. In the same manner, the prediction ranges were also the same as the screen sizes. The data were normalized in the range of 0–1. Missing values were replaced with −1, which is outside the normalized range. ConvNets usually receive images in gray or RGB scale, but the networks can interpret other data types such as go board, shogi board, or chessboard [21]. The ConvNets mathematically calculate the input, whatever the input is; it acts just a series of numbers. Therefore, rather than images, the input of the U-Net used in this study consisted of target tabular data with the specific screen size, previous and next data of the target, and masking matrix for missing data of the target. The number of data input channels was four. Considering it as images, this input becomes an image with N × N pixels and one more dimension than RGB. It was expected that each feature and dimension were considered complex by convolution.

### 2.4. Model Evaluation

To compare the U-Net architecture with existing methodologies, linear interpolation (LI), a feedforward neural network (FFNN), and a long short-term memory (LSTM) were selected. LI is a simple approach to impute missing data; it simply linearly connects intact data. The FFNN is a basic architecture of a neural network algorithm [22]. LSTM is often used for sequence data and exhibits state-of-the-art performance [23]. Since FFNN and LSTM showed reliable accuracies for predicting environmental changes and microclimates in greenhouses, they were selected as comparable models. Owing to structural limitations, the FFNN and LSTM could not have the same input matrices as U-Net (Table 2). The target, previous, and next environmental factors and a loss mask were linearly arranged for the FFNN input. 

The most accurate U-Net and existing models were tested with different all-sensor losses from 10% to 95% to determine the limits of the model robustness by loss percentage (Figure 2b). All losses were randomized using a random number generator with the same random seed. The U-Net and existing models were trained with 30% data loss. Ablation tests with input matrices were also conducted to verify the efficiency of each input component. In all evaluations, the coefficient of determination (R^2^) and root-mean-square error (RMSE) were used as indicators of the accuracy.

## 3. Results

### 3.1. Imputation Accuracies of U-Nets and Other Methods

U-Net_50_ exhibited the highest R^2^ values (Table 3) and the lowest RMSEs (Table 4) for all environmental factors. Among them, the R^2^ value for T_out_ was the highest, while that for CO_2_ was the lowest. In particular, the prediction ability for the missing CO_2_ data was relatively poor, given that the R^2^ values for predicting other environmental factors were near 0.8.

**Figure 4:**
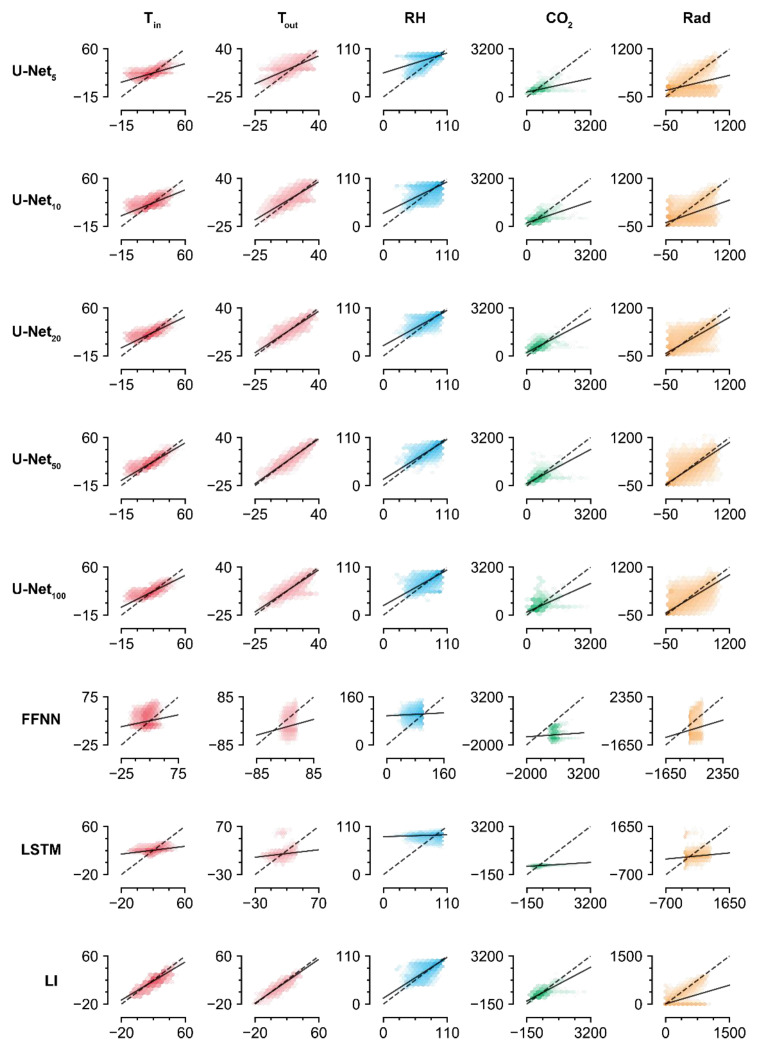
Linear comparison of measured and imputed values. FFNN, LSTM, and LI represent the feedforward neural network, long short-term memory, and linear interpolation, respectively. The subscript represents the screen size. Refer to Table 1 and Table 4 for the abbreviations of environmental factors and for the coefficients and intercepts of regression lines, respectively.

The accuracies of the trained U-Nets increased with screen size, but U-Net_100_ exhibited lower accuracy than U-Net_50_. The values imputed by U-Net_100_ tended to be biased, which could indicate overfitting (Figure 4). Aside from the U-Nets, LI had the highest accuracy for imputation of the missing data. Similar to the U-Nets, the highest prediction accuracy was obtained with T_out_, while the lowest was obtained with Rad. This result contrasts with the high imputation accuracy for radiation obtained by the trained U-Net_50_. The FFNN and LSTM did not exhibit competitive accuracies, although they are deep learning methodologies. According to the R^2^ values, they could not relate the remaining intact data with the missing data.

### 3.2. Model Robustness as Ascertained by the Loss Percentages

**Figure 5:**
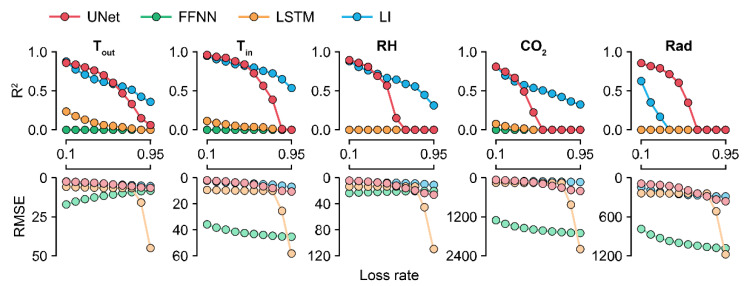
R^2^ and RMSE values ascertained by the loss rates. Colors with low alpha values represent RMSE values. R^2^ values less than zero are depicted as 0.0. FFNN, LSTM, and LI represent the feedforward neural network, long short-term memory, and linear interpolation, respectively. Refer to Table 1 for the abbreviations of environmental factors and the units of RMSE values.

Because U-Net_50_ exhibited the highest accuracy among the U-Nets, it was used to compare the models by their losses. The accuracy of the trained U-Net decreased sharply in the case of CO_2_ (Figure 5). For factors other than CO_2_, U-Net sustained its accuracy at loss rates of <50%. LI sustained its accuracy even with losses of >50%. The RMSE values of the FFNN and LSTM were also changed, although they could not correctly impute the missing data.

### 3.3. Ablations for Key Components of the Input

**Figure 6:**
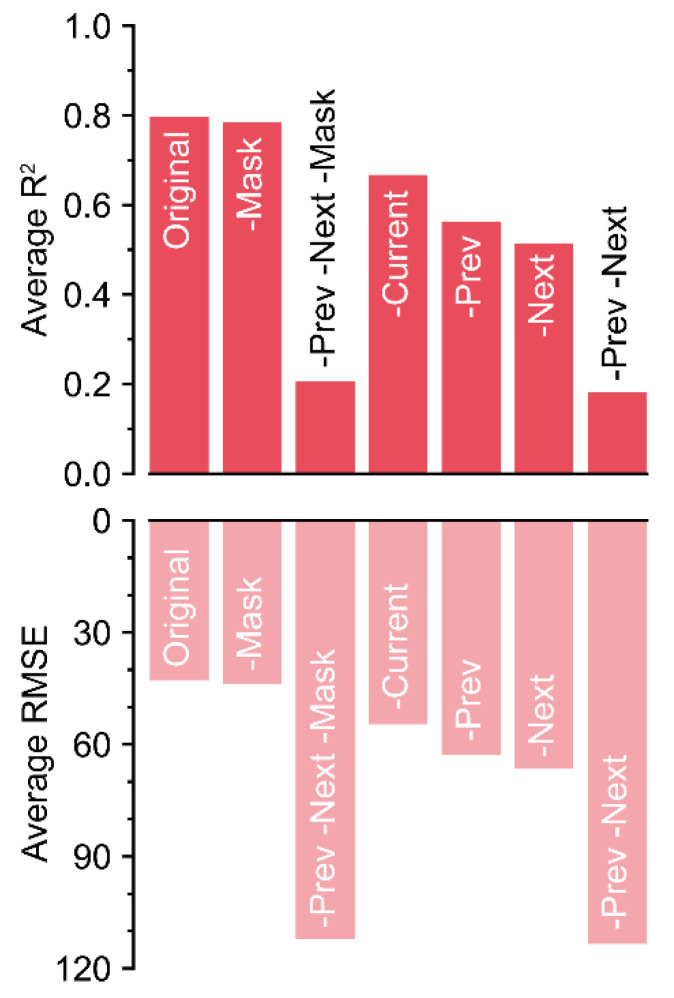
R^2^ and RMSE values ascertained by the loss rates. Colors with low alpha values represent RMSE values.

Because the R^2^ values decreased by almost 0.6 without the previous and next matrices, these matrices were the most influential input components (Figure 6).

The absence of the mask matrix barely reduced the accuracy. Unexpectedly, the current matrix was the next least influential, after the mask matrix. Although the current matrix was the target, the decrease in accuracy was relatively lower compared to that of other input components. In contrast, the trained U-Net could not correctly impute the missing data with only current and mask matrices, although the screen size of 50 included a long-term dataset (>2 days). The magnitude of the decrease could be small, but the exclusion of each component resulted in a decrease in accuracy.

## 4. Discussion

### 4.1. U-Nets

Various screen sizes were compared to evaluate the U-Net architecture for data imputation, and U-Net_50_ exhibited the best performance (Figure 4). That is, U-Net was optimized with a screen size of 50. U-Nets usually handle an image size of >500 × 500 because the input should be compressed and abstracted in multiple layers [18,24]. However, the optimal size was 50 for tabular data, which was 10% of the usual input size of U-Nets. The columns in the images are independent of their size. The small optimal screen size could be due to the strong relation between duplicated columns. Likewise, the low accuracy of the trained U-Net_100_ could be a result of overfitting because the five features of tabular data were too few for this architecture. This could also be due to receptive fields. ConvNet has specific receptive fields according to its architecture, and this could change the way of recognizing input [25]. In this study, all U-Nets had the same receptive fields for model comparison. The same receptive fields could be too small for U-Net_100_, resulting in a narrow view of the inputs. Changing the hyperparameters could improve the performance of the U-Nets. However, U-Net_100_ with the same architecture could be used in other conditions. Environmental factors that can be used for microclimate monitoring have more than five features [26,27]. The optimal screen size could be >50 when more features are in the tabular dataset. In this study with five input features, we found that, even when the number of features is small, the features can be duplicated and imputed.

In the ablations, the absence of each input component caused different decreases in accuracy (Figure 6). A mask with 0 and 1 can be used to train non-image inputs using a ConvNet [16,21]. However, the mask was ineffective for U-Net_50_, as shown by the accuracy being barely changed. In this study, missing values were marked as −1 in the tabular data, which is outside of the normalization range. Therefore, U-Net_50_ could recognize the missing values without the mask matrix. Unlike in the positioning of hostile and friendly markers as in a board game, empty data could be marked as −1. In the case where target positioning is necessary with fully existing real data (e.g., proofreading of tabular data), the mask matrix could be useful.

For the other input components, the trained U-Net_50_ succeeded in imputation of the missing data with comparable accuracy, even without the current matrix. In the case of the current matrix only, U-Net_50_ exhibited the lowest accuracy. That is, the imputation performance was determined by patterns in the previous and next data, not adjacent data. Greenhouse environments exhibit 24-hour patterns, although they may vary by season [28,29]. Therefore, a screen size of 20 can yield a high accuracy. However, all-sensor losses were designed to be 48 h. It seems that the screen size of 50 exceeded the length of all-sensor losses; therefore, it could be the optimal length. In generalization of the U-Net, high accuracy will be obtained only when it matches the appropriate pattern range of tabular data.

However, because the accuracy slightly decreased without the current matrix, the current matrix was not inoperative. The U-Net somewhat weighted existing values adjacent to missing values, like LI. Although it could be a small decrease, all components were likely used correctly because ablation of all components resulted in a decrease in accuracy.

**Figure 7:**
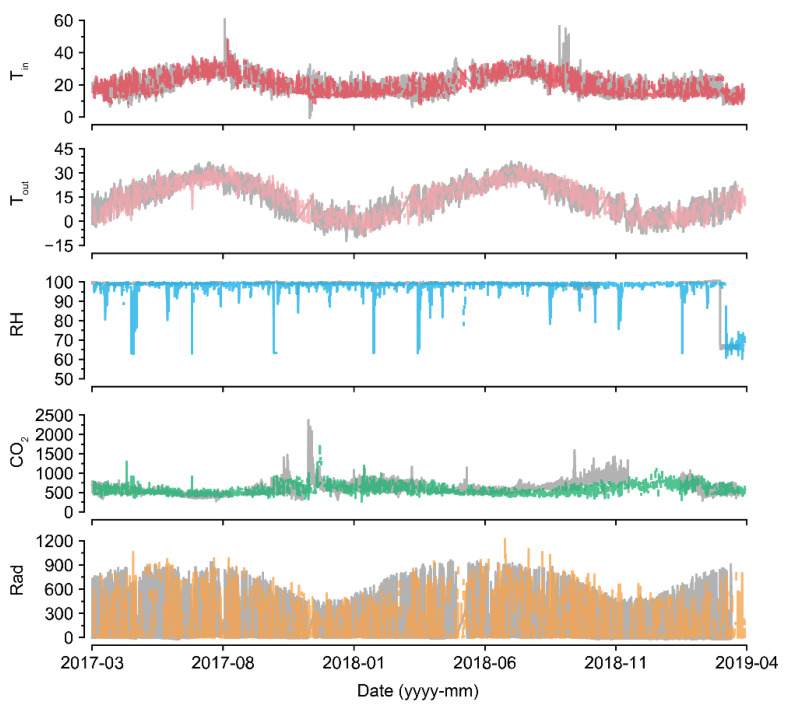
Long-term examples of a recovered environmental dataset (GH24 in Figure 2a; 50% data loss). Gray and colored lines represent intact raw data and imputed data, respectively. Refer to Table 1 for the units of environmental factors.

**Figure 8:**
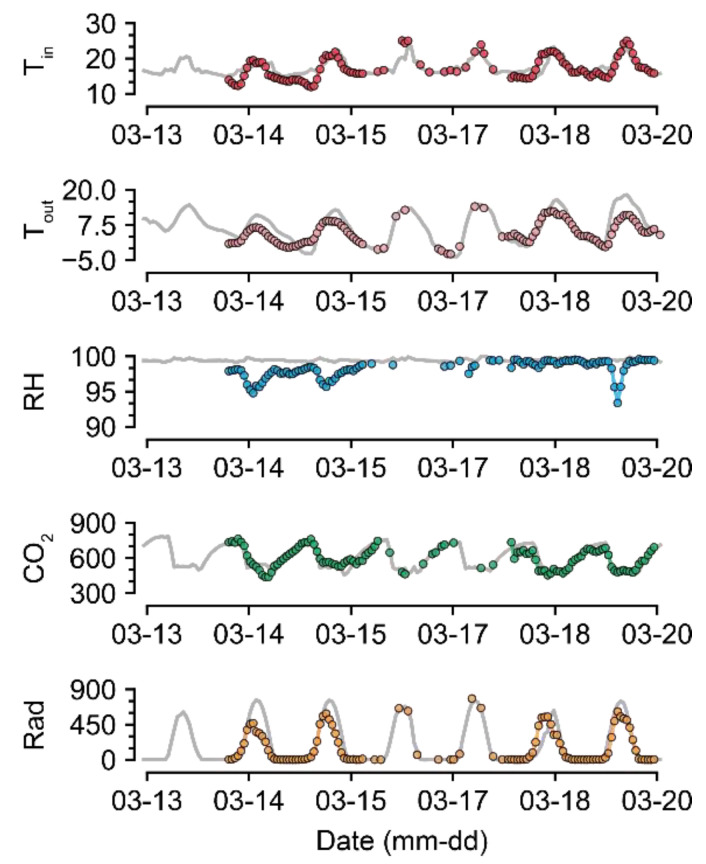
Short-term examples of a recovered environmental dataset (GH24; 50% data loss). Gray and colored lines represent intact raw data and imputed data, respectively. Refer to Table 1 for the units of environmental factors.

For U-Net_50_, even when almost half of the data were missing, the accuracy was maintained to some extent (Figure 5). Compared to the intact data, the imputation was also reasonable (Figure 7 and Figure 8). The sustained accuracy of the U-Net could be due to the fact that the model learned from the training dataset [30]. The model can learn more patterns and increase its accuracy by continuing training in the same environment.

### 4.2. Other Models

LI is a method used to simply splice the nearest intact data. Although it was evident that LI did not properly impute all-sensor losses, which were two days long, it yielded comparable accuracy (Table 3). Therefore, a new type of metric is needed to clearly compare models in missing data imputation.

The low accuracies of the FFNN and LSTM could result from clumsiness in the input [31]. They exhibited comparable accuracies for agricultural estimations or predictions [32,33,34]. The inputs of the FFNN and LSTM included the previous, next, and mask matrices for comparison with the U-Nets. The matrices were expected to give more information about the missing values and the data pattern, but FFNN and LSTM could not interpret the relations between the input features. Since the data imputation task was not a simple prediction, it seemed to require more complicated interpretation of the input and the output. U-Nets succeeded in extracting the importance of each “pixel”, but FFNN and LSTM seemed to be biased by missing values.

Because the FFNN and LSTM are machine learning methodologies, changes in their accuracies yielded by different loss rates imply that the models learned something from the training (Figure 5). However, the FFNN could not interpret a long period of the previous and next data. LSTM cannot convolute target tabular data because it reads the data sequence by sequence. Therefore, a wide range of the datasheet should be considered, and all tabular data should be calculated beyond the sequences.

### 4.3. Variation in Input Environmental Factors

U-Net_50_ and LI exhibited the highest accuracy for T_out_ among the five input factors. T_out_ was also less affected by the loss percentages. That is, T_out_ could be a simple factor to impute. The chosen greenhouses were in the same climate conditions; thus, the individual datasets could share a tendency with respect to T_out_. Most importantly, T_out_ was not a factor controlled by the grower. Therefore, the pattern could be easily extracted by the models.

Meanwhile, the imputation of missing T_in_ did not exhibit as high an accuracy as in the case of T_out_. The models could not impute T_in_, although this factor also has somewhat constant patterns because the internal environments of greenhouses are controlled to be within specific ranges [4]. Neural network algorithms yielded high performance in previous studies [35,36]. Unlike T_out_, T_in_ could be affected by different grower strategies [37]. Therefore, the datasets did not seem to share the changing patterns; thus, the models could not impute the missing T_in_ with as high an accuracy as for T_out_.

U-Net_50_ exhibited a higher variance when imputing the RH than when imputing other environmental factors, even though the measured RH was sustained at almost 100% (Figure 7). In greenhouses, the RH can be sustained at 100%, but it tends to drop and be restored immediately after sunrise because of thermal screens and ventilation [38,39]. RH sensors have been reported to have high error and failure rates [40]. Therefore, the measured values could be incorrect. Consequently, it seems that U-Net_50_ can be used for proofreading of error data, as well as for the imputation of missing data. Based on the flexibility of the deep learning algorithm, the U-Nets could be remodeled with only a few input and output changes.

In terms of CO_2_, the control strategy barely showed a pattern (Figure 7). In particular, the imputation accuracy of CO_2_ declined with an increase in the loss rate (Figure 5). That is, the relationship between CO_2_ and other environmental factors could be weak. This could be due to the control strategies of CO_2_. CO_2_ fertilization is usually conducted empirically [41,42]. In this study, greenhouses used manual CO_2_ fertilization, except for some advanced farms. Therefore, the models could not find definite patterns of CO_2_ changes. In this case, control data could be used to improve the robustness [43]. However, U-Net_50_ exhibited adequate accuracy for CO_2_ imputation, although it was relatively lower than the accuracy for other environmental factors.

LI failed to impute Rad, but U-Net did so adequately (Table 3). This seems to be due to the existence of nighttime data, as LI simply splices the intact values; thus, the zero Rad at nighttime could cause high errors in imputing Rad. U-Nets could distinguish day and night regardless of the position of the input screen, although Rad was somewhat overestimated or underestimated. It can be said that U-Net could learn specific patterns in tabular data that LI could not, as LI does not have model training.

## 5. Conclusions

In this study, U-Net architectures were evaluated from the perspective of data imputation based on missing tabular data from 27 greenhouses. The trained U-Net exhibited an acceptable accuracy (average R^2^ = 0.80), and the highest accuracy was obtained with a screen size of 50. Among the other models tested, LI exhibited comparable performance. The FFNN and LSTM could not be properly trained. Based on the accuracies for imputing five environmental factors, U-Net seemed to adequately learn the change patterns in the tabular data, although U-Nets are usually used for images. The trained U-Nets sustained their robustness with increasing loss rate, demonstrating their usefulness for tabular data imputation with short-term and long-term losses at the same time.

## Figures and Tables

**Table 1:** Ranges of environmental data used for the experiment.

Environmental Factor	Abbreviation	Range
Internal temperature (°C)	T_in_	5.3 to 60.3
External temperature (°C)	T_out_	−21.2 to 38.0
Internal relative humidity (%)	RH	19.4 to 101.3
Internal CO_2_ concentration (μmol mol^−1^)	CO_2_	1.7 to 2999.0
Radiation (W m^−2^)	Rad	0.0 to 1669.9.

**Table 2:** Architectures of the compared models. Layer parameters are denoted as type of layer and number of nodes in the layer (number of trainable parameters). FFNN and LSTM represent a feedforward neural network and long short-term memory, respectively.

Model	FFNN	LSTM
Input size	1 × 20	100 × 20
Layers	Dense 64 (384)	BiLSTM 64 (43,520)
	Dense 64 (4160)	BiLSTM 64 (98,816)
	Dense 5 (325)	Dense 5 (645)
Output size	1 × 5	100 × 5

**Table 3:** R^2^ values of the models. The boldface values are the highest R^2^ values for each factor. FFNN, LSTM, and LI represent the feedforward neural network, long short-term memory, and linear interpolation, respectively. The subscript represents the screen size. See Table 1 for the abbreviations of environmental factors.

	U-Net_5_	U-Net_10_	U-Net_20_	U-Net_50_	U-Net_100_	FFNN	LSTM	LI
T_in_	0.32	0.45	0.67	**0.80**	0.66	−3.34	0.13	0.71
T_out_	0.49	0.69	0.87	**0.92**	0.85	−14.81	0.07	0.88
RH	0.33	0.49	0.75	**0.81**	0.57	−1.74	−0.04	0.76
CO_2_	0.23	0.23	0.21	**0.66**	0.23	−85.32	0.03	0.62
Rad	0.22	0.41	0.69	**0.79**	0.68	−14.25	0.01	0.17

**Table 4:** Root-mean-square error (RMSE) values of the models. The boldface values are the lowest RMSE values for each factor. FFNN, LSTM, and LI represent the feedforward neural network, long short-term memory, and linear interpolation, respectively. The subscript represents the screen size. Refer to Table 1 for the abbreviations of environmental factors.

	U-Net_5_	U-Net_10_	U-Net_20_	U-Net_50_	U-Net_100_	FFNN	LSTM	LI
T_in_	5.44	4.90	3.79	**2.95**	3.88	13.82	6.18	3.57
T_out_	7.16	5.57	3.61	**2.81**	3.83	39.98	9.69	3.54
RH	11.02	9.63	6.75	**5.91**	8.77	22.25	13.70	6.58
CO_2_	141.33	141.61	143.00	**93.19**	141.17	1494.73	158.64	99.42
Rad	210.03	182.91	132.40	**109.40**	134.71	927.42	238.57	216.69

## Data Availability

Data is contained within the article.
